# New‐generation anticancer drugs and medication‐related osteonecrosis of the jaw (MRONJ): Late onset 3 years after ipilimumab endovenous administration with a possible role of target therapy

**DOI:** 10.1002/ccr3.3418

**Published:** 2020-12-02

**Authors:** Agostino Guida, Francesco Perri, Franco Ionna, Paolo A. Ascierto, Antonio M. Grimaldi

**Affiliations:** ^1^ Maxillo‐facial and ENT Surgery Unit INT – IRCCS “Fondazione G. Pascale” Naples Italy; ^2^ Head & Neck/Thyroid Medical Oncology Unit INT – IRCCS “Fondazione G. Pascale” Naples Italy; ^3^ Melanoma, Oncological Immunotherapy and Innovative Therapies Department INT – IRCCS “Fondazione G. Pascale” Naples Italy

**Keywords:** bisphosphonate‐associated osteonecrosis, ipilimumab, medically compromised patients, medication‐related osteonecrosis of the jaw, target therapy

## Abstract

Association of immunotherapy and/or chemotherapy and/or targeted therapy, in sequence or as single therapies, may induce osteonecrosis of the jaw. Multidisciplinary team management of these patients should be provided.

## Graphical abstract

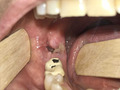

## 1 INTRODUCTION

The number of medication which may cause osteonecrosis of the jaws is increasing. Up until now, Ipilimumab has been associated with MRONJ only two times in literature. A woman underwent endovenous chemotherapy with ipilimumab in 2015 for metastatic melanoma. In 2018, while she was undergoing target therapy (vemurafenib + cobimetinib), after wisdom tooth extraction, she developed MRONJ. She was successfully treated with medical therapy alone. Ongoing target therapy may have played a role in MRONJ late onset. Caution and vigilance in dental management of patients treated with novel MRONJ‐related chemotherapy are needed. A multidisciplinary evaluation is advised.

First reported cases of nonhealing‐exposed bone in the maxillofacial region were recognized by oral and maxillofacial surgeons in patients treated with intravenous (IV) bisphosphonates (BP).^1^ During 2004, Novartis, manufacturer of pamidronate (Aredia) and zoledronic acid (Zometa)—two IV BPs—labeled this product as at risk for osteonecrosis of the jaws (ONJ).^2^ Consequently, the subsequent year a warning followed for all BP drug class to be at risk for ONJ, which was renamed as bisphosphonate‐related ONJ (BRONJ).^3^

Since then, other BPs and medications from other classes have been related to the development of ONJ, including denosumab (humanized monoclonal antibody blocking the activation of receptors for nuclear factor κβ ligand), bevacizumab (humanized monoclonal antibody), and antiangiogenic medications—sunitinib (tyrosine kinase inhibitor).^4^, ^5^, ^6^, ^7^, ^8^, ^9^, ^10^ Additionally, case reports have indicated possible association between ONJ and azacitidine, imatinib, everolimus, ziv‐aflibercept, ipilimumab, and tocilizumab.^11^, ^12^, ^13^, ^14^, ^15^, ^16^, ^17^, ^18^ With the advent of these new classes of medications, the condition is now more aptly known as medication‐related osteonecrosis of the jaw (MRONJ).^1^ Both pathogenesis and associated risk factors not fully comprehended. This majorly reflects on its therapy as indications for surgery (sequestrectomy/curettage with possible reconstruction) vs antibiotic medical therapy (3 g amoxicillin + 1.5 metronidazole per os per day for at least 2 weeks) are still unclear and so great effort is also put in studying and developing complementary treatments, including application of platelet concentrates, ozone therapy, and laser treatment, in order both to prevent MRONJ and to improve healing after surgical treatment of such lesions.^19^

Uncertainty increases both for diagnosis and treatment especially for non‐BP drugs related to ONJ, which may have very few cases reported in literature, such as ipilimumab.

Ipilimumab is a monoclonal antibody directed against the CTLA4 receptor, present on activated T lymphocytes. The resulting binding causes an increase of lymphocyte T activity directed against melanoma cells, which are therefore destructed. The antibody is administered intravenously at a dose of 3 mg/kg every 3 weeks, for 4 cycles. Approval of ipilimumab was based on a randomized three‐arm phase III study which compared ipilimumab with a vaccine therapy (gp100) and with their combination,^20^ showing improved overall survival in patients undergoing Ipilimumab. Ipilimumab is associated with the risk of immune‐related side effects; sixty percent of immune‐related adverse events were recorded in the study population. Approximately 15% of patients experienced grade 3 or 4 adverse events. Dermatitis was the most frequent immune‐related event, and diarrhea, the most dangerous (perforation risk if not promptly treated); severe cases should be treated with high‐dose corticosteroids.

In present scientific literature, there are two reported cases of MRONJ onset in patients treated with ipilimumab alone and 1 in a patient treated with concomitant denosumab + ipilimumab.^12^, ^17^ MRONJ onset was in all cases during ipilimumab therapy or shortly after the conclusion of it.

In this case report, we describe MRONJ occurred 3 years after the conclusion of treatment with ipilimumab.

## 2 CASE PRESENTATION

**FIGURE 1:**
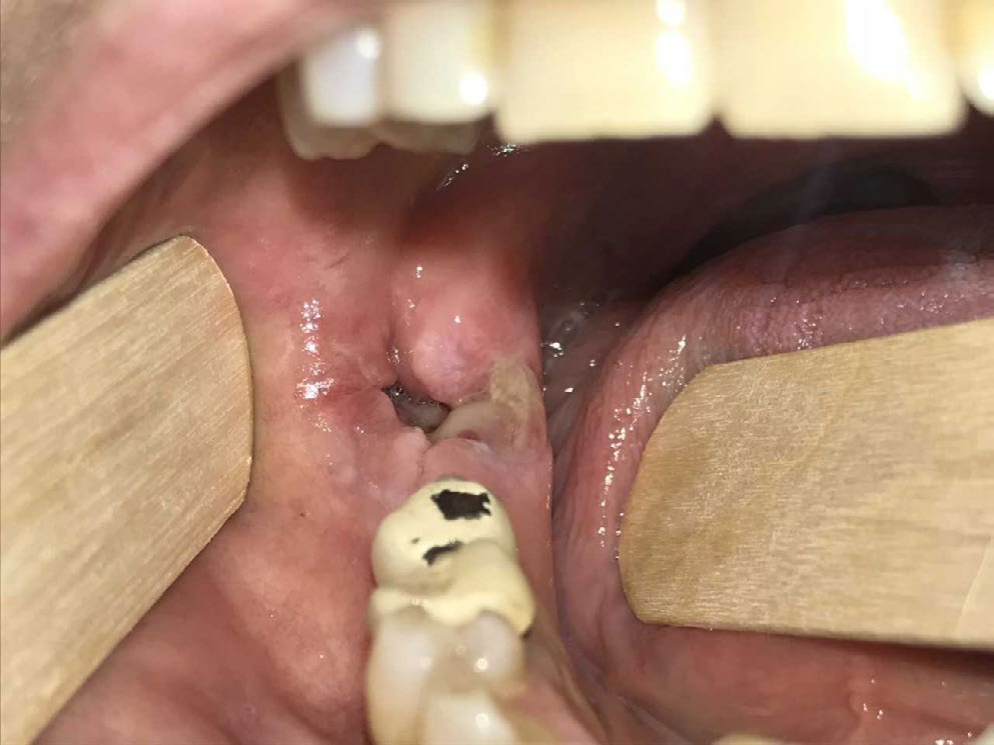
Intraoral inspection revealed the nonhealing alveolus

In November 2018, a 58 year‐old‐woman with BRAF‐mutated metastatic melanoma, treated at the immunotherapy unit of our institute, was referred to our oral pathology outpatient clinic. During a regular follow‐up visit, the patient reported that she was experiencing severe pain in the oral cavity for 4 months due to a nonhealing alveolus, after the extraction of the lower right third molar. During this time, the patient had been treated by her dentist for an alveolar osteitis (AO, dry sockets) with 1‐week cycle of amoxicillin + clavulanic acid (2.25 + 0.75 g/d per os) and chlorhexidine 0.2% mouthwash daily socket irrigation. She referred that she had repeated this therapy three times during those months, continuing chlorhexidine 0.2% mouthwash daily socket irrigation among the antibiotic cycles. She referred that, occasionally, she also underwent application of zinc oxide eugenol in her alveolus. Furthermore, the patient had used chlorhexidine 0.2% mouth rinse since day before the extraction. Every treatment tried up until now had been unsuccessful. She had no extra‐oral sign of swelling nor of ongoing abscess. Intraorally, clinical inspection confirmed the presence of a nonhealed alveolar socket; the bottom and the walls of the alveolus were clearly visible, made of nonvascularized nonsuppurated bone, surrounded by swollen mucosa (Figure 1).

**FIGURE 2:**
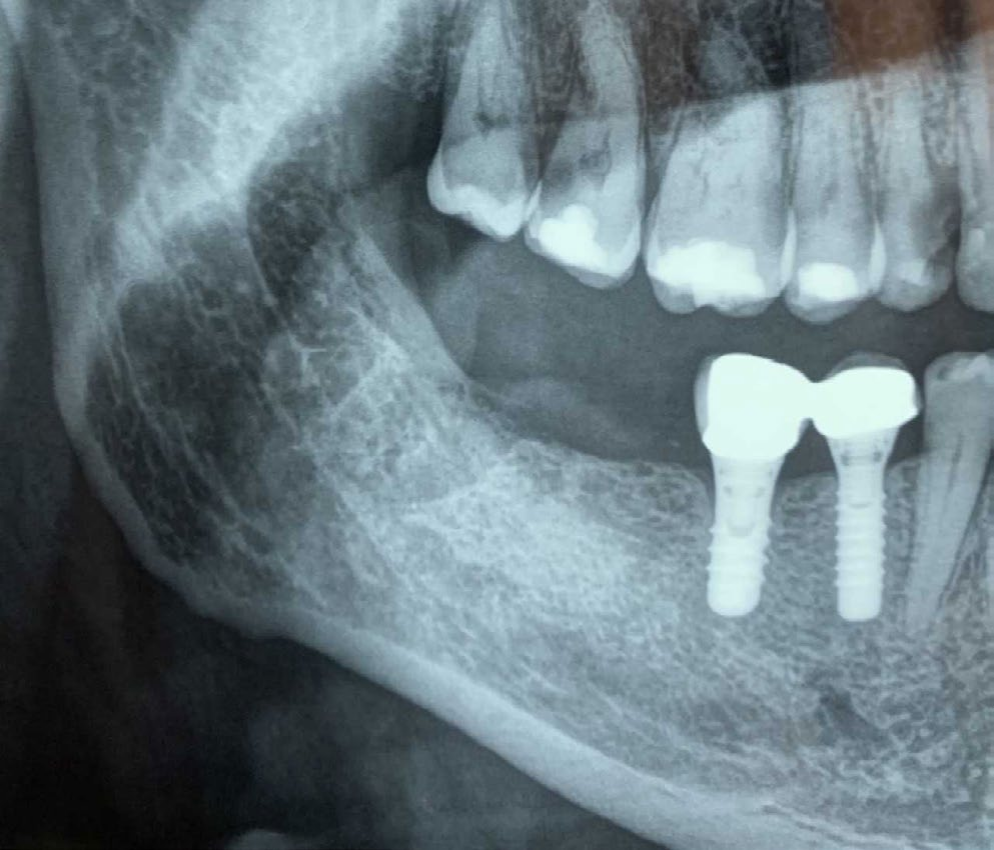
Orthopantomography (OPT) exhibited from the patient during the first visit, revealing the nonhealing alveolus 4 mo after tooth extraction

She exhibited a 3‐day‐old orthopantomography (OPT), which showed radiographic sign of a nonhealed alveolus (Figure 2).

**FIGURE 3:**
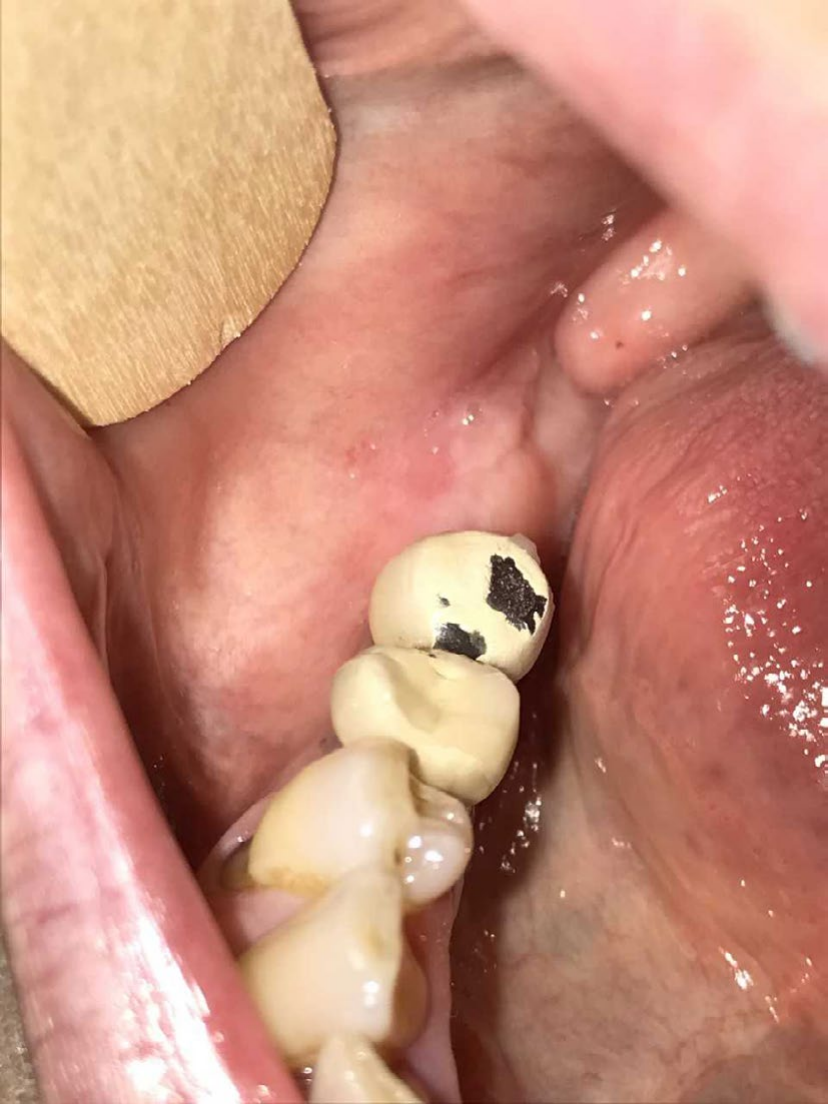
Complete clinical resolution after 4 wks of antibiotic/disinfectant therapy (amoxicillin + metronidazole −3 + 1.5 g per os/die‐ and chlorhexidine 0.2% mouth rinse)

**FIGURE 4:**
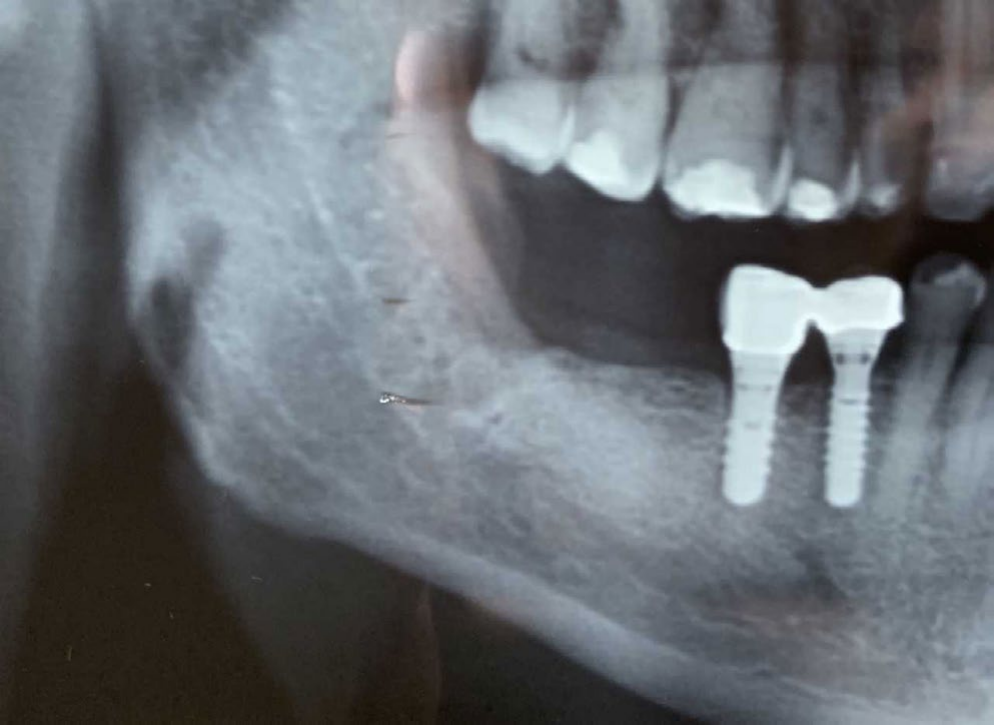
Radiographic appearance at OPT after 6 mo of follow‐up, showing complete bone healing

Her anamnesis was carefully harvested. The patient underwent a surgical resection of a cutaneous melanoma in 2009. Then, in 2015, for lung progression of disease, she was treated with ipilimumab (3 mg/kg mg iv, every 3 weeks for 4 cycles) with complete remission of the disease. During the follow‐up, in 2017 the patient had hepatic progression, and so, due to the presence of the BRAF mutation, she started the treatment with dabrafenib + trametinib (300 + 2 mg per os/die). Due to the G. 3 toxicity (fever) experienced by the patient, the treatment was stopped and was replaced with vemurafenib + cobimetinib (vemurafenib: 1920 per os/die for 3 months, then 1440 mg per os/die; cobimetinib: 60 mg per os/die for 3 weeks then 1‐week pause), still ongoing. She was then taking 1440 mg vemurafenib + 60 mg cobimetinib per os/die at the moment of her tooth extraction. She had no history of smoking nor head and neck radiotherapy. Among all the medications she had undergone, ipilimumab was the only one that has been related to MRONJ.^12^, ^17^ Staging of the MRONJ was thus performed; it was evaluated to be a “stage 2 MRONJ” according to the AAOMS classification, showing “Exposed and necrotic bone(…) with evidence of infection, (…) symptomatic.”^1^ She was thus treated accordingly, starting a treatment with amoxicillin + metronidazole (3 + 1.5 g per os/die) and chlorhexidine 0.2% mouth rinse twice a day; paracetamol (1 g per os) was prescribed for pain control. During the 2‐week follow‐up visit, the patient showed clinical improvement. She referred the ejection of a 10 × 5 mm bone sequestrum after 6 days of therapy and that her symptoms had therefore disappeared. The clinical examination still highlighted an incomplete alveolar healing. Two additional weeks of therapy were prescribed and, after that, the patient obtained a complete healing of the defect (Figure 3). Treatment for the MRONJ was stopped, and the patient was regularly followed up monthly. After 6 months, a new OPT showed complete healing of the alveolus (Figure 4).

## 3 DISCUSSION

Several new medications have been added to the potential cause of MRONJ drug list. Among these, ipilimumab has been reported in three published clinical cases as a possible cause of MRONJ, two as single therapy and one in association with denosumab.^12^, ^20^ As far as literature reports, MRONJ onset was in all cases during ipilimumab therapy or shortly after the conclusion. Our patient suspended the treatment with ipilimumab 3 years before. Dabrafenib, trametinib, vemurafenib, and cobimetinib—the other chemotherapy drugs taken by the patient—have never been reported as possible cause of MRONJ.

Therefore, diagnosis was the first issue we encountered in the management of this case. For the symptoms and the clinical presentation, the differential diagnosis was between alveolar osteitis (dry socket, AO), MRONJ, and osteoradionecrosis (ORN). ORN was the first possibility to be discarded as the patients had no history of head and neck radiotherapy. AO was carefully taken into account prior to start any therapy. The clinical and radiographic appearance was indeed compatible with such disease which is defined as “postoperative pain in and around the extraction site, which increases in severity at any time between one and 3 days after the extraction, associated with a partially or totally disintegrated blood clot within the alveolar socket, with or without halitosis.”^21^ Still, as a matter of fact, most recent meta‐analyses show that AO therapy should be more symptomatic^21^, ^22^ rather than therapeutic, as there is no full comprehension of its pathogenesis and, above all, it is considered as a “self‐limiting” disease. The antibiotic therapy prescribed by the dentist of the patient resulted useless, and no improvement was observed after 4 months even with topic injection of chlorhexidine and zinc oxide eugenol, which are reported between the most successful treatment for AO.^21^, ^22^ As reported in literature, after the diagnosis, AO, regardless of the therapy, tends to remit in a period of days or weeks, most commonly,^22^ while there is no report in scientific literature of AO persisting for several months. AO was thus discarded in the differential diagnosis process.

Furthermore, the presence of a bone sequestrum related to a nonhealing postextraction socket, not visible at the first inspection but ejected during antibiotic therapy, is an event more compatible with MRONJ rather than AO. About the therapy administered, it must be underlined that no guideline exists yet. Consensus conferences^1^ advise to begin with antibiotic therapy and then, in case of partial/no response, re‐evaluate the patient for surgery. In our case, antibiotic therapy was administered as first choice and we reached complete healing. Yet, literature^23^ warns that partial/no response to antibiotic therapy is a common event, and so is the necessity to complete the therapeutic pathway with surgical approach in order to reach complete healing. Patients must be thus followed up carefully.

As reported before, among the various anticancer therapy agents administered to the patient, ipilimumab was the only drug that could be related to MRONJ. Ipilimumab was approved by the US Food and Drug Administration in March 2011 as an immunotherapy for the management of advanced (unresectable or metastatic) melanoma patients.^12^ Ipilimumab is a humanized monoclonal antibody against cytotoxic T lymphocyte–associated antigen‐4 (CTLA‐4). CTLA‐4 is expressed both in activated T cells and in suppressor T‐regulatory cells, binding to antigen‐presenting cells and therefore diminishing T‐cell responses. The block of the CTLA‐4 is able to improve the antitumor responses of activated T cells. The result is a significant incremented survival in patients with metastatic melanoma undergoing ipilimumab.^18^, ^24^ The immune response induced by ipilimumab with only 4 cycles of treatment (about 2 months of therapy) can persist for many years, inducing a kind of vaccination against metastatic melanoma. In literature, there are reported cases of ipilimumab‐related ONJ occurred during or shortly after the end of the systemic therapy. The authors suggested that that Ipilimumab may have been involved in the process of bone necrosis by empowering the number of systemic activated T‐cell presence. CTLA4‐deficient activated T cells have been shown to be associated with osteonecrosis, as activated T cells may ignite osteoclastogenesis via osteoprotegerin ligand, resulting in bone loss.^25^ Trauma from regular oral activity or oral surgery (eg, tooth extraction) could increase the demand for this vulnerable bone to mend itself, resulting in localized bone necrosis.^12^

Ipilimumab is known to have a 14.7‐day blood half‐life^26^ while the patient described in our case had completed Ipilimumab treatment 3 years before. As we have seen, the real advantage of the drug is in the long‐term efficacy with about 20% of patients alive at 5, 7, and 10 years after treatment completion. This long‐term efficacy is due to the immune responses induced by checkpoint inhibitors. Still, just like the anticancer effects, side effects can last for many years.^12^ It is conceivable that, similarly to pruritus, diarrhea, vitiligo, hepatitis, and endocrinopathies, MRONJ may be also a late side effect under certain circumstances. We suggest that the MRONJ onset may have been co‐caused by the ongoing target therapy (vemurafenib + cobimetinib) of the patient. The effect of BRAF and MEK inhibitors in BRAF‐mutant melanoma can lead to an immune‐stimulating microenvironment by enhancing expression of immune‐stimulating molecules and cytokines, reducing immunosuppressive cell populations, and decreasing immunosuppressive cytokines. The cell damage to the tumor by the target therapy may have induced a tumor‐antigen spreading, restimulating T‐cell activity whose response had been increased and modulated by the effect of ipilimumab. Moreover, it has been demonstrated that anti‐BRAF therapy enhances the reactivity and cytotoxicity of T cells.^27^, ^28^ The re‐activation of such empowered T‐cell clones may have lead the patient into a window of time in which she was at risk for MRONJ, similarly to when the patient was on treatment with ipilimumab.

## 4 CONCLUSIONS

In addition to well‐known medications, MRONJ may be a major adverse reaction to several new‐generation anticancer drugs. These drugs may have unexpected mechanisms, being their pharmacodynamic not fully comprehended up until now. Even if this paper reports of a single event—in addition to the few other cases reported in literature of ipilimumab MRONJ, the authors recommend caution and strict vigilance in the dental management of patients treated with novel chemotherapy drugs, reported to be at risk for MRONJ. Multidisciplinary evaluation is thus strongly advised; cooperation between the oncologist and the dentist/oral and maxillofacial surgeon may help in taking the best decision in the patient's interest, ensuring the best possible result in the management of relatively recent drugs, which may cause unpredictable side effects. The administration of the prophylactic antibiotic protocol (amoxicillin + metronidazole; 3 + 1.5 g per os/die) may be arranged in accordance between the surgeon and the oncologist, with the best possible evaluation of both oral and systemic conditions. Such cooperation may reduce the occurrence of adverse events which, as we have shown in our paper, may result in patient's discomfort and pain. Further studies are needed on a large number of cases, in order to fully understand the relation between ipilimumab and MRONJ, and the possible interference of target therapy.

## 5 ETHICS APPROVAL AND CONSENT TO PARTICIPATE

Not applicable.

## 6 CONSENT FOR PUBLICATION

Not applicable.

## CONFLICT OF INTEREST

The authors declare that they have no competing interests.

## AUTHOR CONTRIBUTIONS

AG and AMG: drafted the manuscript. FP: revised the manuscript. FI and PAA: performed scientific supervision.

## Data Availability

The datasets used and analyzed during the current study are available from the corresponding author upon reasonable request.
